# Polygenic risk for anxiety influences anxiety comorbidity and suicidal behavior in bipolar disorder

**DOI:** 10.1038/s41398-020-00981-5

**Published:** 2020-08-24

**Authors:** Fabiana L. Lopes, Kevin Zhu, Kirstin L. Purves, Christopher Song, Kwangmi Ahn, Liping Hou, Nirmala Akula, Layla Kassem, Sarah E. Bergen, Mikael Landen, Andre B. Veras, Antonio E. Nardi, Bipolar Genome Study Consortium (BiGS), Ney Alliey-Rodriguez, Judith A. Badner, Wade Berrettini, William Byerley, William Coryell, David W. Craig, Howard J. Edenberg, Tatiana Foroud, Elliot S. Gershon, Tiffany A. Greenwood, Yiran Guo, Brendan J. Keating, Daniel L. Koller, William B. Lawson, Chunyu Liu, Pamela B. Mahon, Melvin G. McInnis, Sarah S. Murray, John L. Nurnberger, Evaristus A. Nwulia, Corrie B. Panganiban, John Rice, Nicholas J. Schork, Erin N. Smith, Peng Zhang, Sebastian Zöllner, Fernando S. Goes, John R. Kelsoe, Caroline M. Nievergelt, James B. Potash, Tatyana Shekhtman, Paul D. Schilling, Peter P. Zandi, Francis J. McMahon

**Affiliations:** 1 Intramural Research Program, National Institute of Mental Health, National Institutes of Health, US Department of Health & Human Services, Bethesda, MD USA; 2 King’s College London, Social, Genetic and Developmental Psychiatry Centre, Institute of Psychiatry, Psychology & Neuroscience, London, UK; 3 Department of Medical Epidemiology and Biostatistics, Karolinska Institutet, Stockholm, Sweden; 4 Institute of Neuroscience and Physiology, Department of Psychiatry and Neurochemistry, the Sahlgrenska Academy, University of Gothenburg, Gothenburg, Sweden; 5 Institute of Psychiatry, Federal University of Rio de Janeiro, Rio de Janeiro, RJ Brazil; 6 Department of Psychiatry and Behavioral Neuroscience, University of Chicago, Chicago, IL USA; 7 Department of Psychiatry, University of Pennsylvania, Philadelphia, PA USA; 8 Department of Psychiatry, University of California at San Francisco, San Francisco, CA USA; 9 University of Iowa Hospitals and Clinics, Iowa City, IA (Coryell) USA; 10 The Translational Genomics Research Institute, Phoenix, AZ USA; 11 Department of Biochemistry and Molecular Biology, Indiana University School of Medicine, Indianapolis, IN USA; 12 Department of Medical and Molecular Genetics, Indiana University School of Medicine, Indianapolis, IN USA; 13 Department of Psychiatry, University of California San Diego, San Diego, CA USA; 14 Center for Applied Genomics, Children’s Hospital of Philadelphia, Abramson Research Center, Philadelphia, PA USA; 15 Institute for Translational Medicine and Therapeutics, School of Medicine, University of Pennsylvania, Philadelphia, PA USA; 16 Dell Medical School, University of Texas at Austin, Austin, TX USA; 17 Department of Psychiatry, SUNY Upstate Medical University, Syracuse, NY USA; 18 Department of Psychiatry and Behavioral Sciences, John Hopkins School of Medicine, Baltimore, MD USA; 19 Department of Psychiatry, University of Michigan, Ann Arbor, MI USA; 20 Department of Pathology, University of California San Diego, La Jolla, USA; 21 Department of Psychiatry, Indiana University School of Medicine, Indianapolis, IN USA; 22 Department of Psychiatry and Behavioral Sciences, Howard University Hospital, Washington, DC USA; 23 Department of Psychiatry, Washington University School of Medicine in St. Louis, St. Louis, MO USA; 24 University of California, San Diego, La Jolla, CA USA; 25 J. Craig Venter Institute, La Jolla, CA USA; 26 Scripps Genomic Medicine & The Scripps Translational Sciences Institute (STSI), La Jolla, CA USA; 27 Department of Pediatrics and Rady’s Children’s Hospital, School of Medicine, University of California San Diego, La Jolla, CA USA; 28 Department of Computational Medicine and Bioinformatics, University of Michigan, Ann Arbor, MI USA; 29 Department of Molecular Medicine and Surgery, Karolinska Institutet, Stockholm, Sweden; 30 Center for Molecular Medicine, Karolinska University Hospital, Stockholm, Sweden; 31 Department of Mental Health, Johns Hopkins Bloomberg School of Public Health, Baltimore, MD USA

**Keywords:** Clinical genetics, Bipolar disorder

## Abstract

Bipolar disorder is often comorbid with anxiety, which is itself associated with poorer clinical outcomes, including suicide. A better etiologic understanding of this comorbidity could inform diagnosis and treatment. The present study aims to test whether comorbid anxiety in bipolar disorder reflects shared genetic risk factors. We also sought to assess the contribution of genetic risk for anxiety to suicide attempts in bipolar disorder. Polygenic risk scores (PRS) were calculated from published genome-wide association studies of samples of controls and cases with anxiety (*n* = 83,566) or bipolar disorder (*n* = 51,710), then scored in independent target samples (total *n* = 3369) of individuals with bipolar disorder who reported or denied lifetime anxiety disorders or suicidal attempts in research interviews. Participants were recruited from clinical and nonclinical settings and genotyped for common genetic variants. The results show that polygenic risk for anxiety was associated with comorbid anxiety disorders and suicide attempts in bipolar disorder, while polygenic risk for bipolar disorder was not associated with any of these variables. Our findings point out that comorbid anxiety disorders in bipolar disorder reflect a dual burden of bipolar and anxiety-related genes; the latter may also contribute to suicide attempts. Clinical care that recognizes and addresses this dual burden may help improve outcomes in people living with comorbid bipolar and anxiety disorders.

## Introduction

Anxiety and bipolar disorder (BP) are highly comorbid conditions^1,2^, but the basis of this comorbidity is uncertain. There is some overlap in diagnostic criteria, but this is not substantial.

It is possible that both conditions share environmental risk factors but these do not appear to be much more common in people with comorbid anxiety and BP than in those with BP alone^1^. Familial co-aggregation of anxiety and bipolar disorder^3,4^ suggests shared genetic risk factors, but this has not been tested using molecular genetic methods.

Comorbid anxiety has important clinical implications for people living with BP. Several studies have reported unfavorable outcomes in BP with comorbid anxiety, including more frequent mood episodes^5,6^, more severe depressive episodes^7^, higher rates of substance abuse^6^, less favorable treatment response^6,8,9^, and increased suicide attempts^10–12^. The United States National Comorbidity Survey found that the diagnosis of anxiety disorder is particularly frequent among individuals with suicide attempts^13^. A deeper understanding of the comorbidity between anxiety and BP could shed light on these clinical problems and could in future point toward new treatment approaches.

BP is a highly heritable disease (~80%), and many genome wide association studies (GWAS) have been published, together finding close to 40 replicated genetic loci associated with the disorder^14,15^. Molecular genetic studies of anxiety are still relatively uncommon^16^, with many studies having small samples and as a result inconsistently replicated results. Two recent large-scale GWAS of anxiety and anxiety disorders have made considerable progress in identifying robust genetic risk factors^17,18^.

Both studies found that anxiety is a highly polygenic trait, like most other psychiatric conditions, with thousands of associated alleles that each contribute a small share to the total risk. Polygenic risk scores (PRS) are a powerful tool that capitalize on this cumulative genetic risk. One way in which these scores can be used is to identify shared genetic liability—if people with one disorder have a high polygenetic risk score for a second disorder it may indicate a shared genetic liability. In the field of psychiatric disorders, the PRS was initially used by Purcell et al. in a study of schizophrenia^19^. The PRS approach has since been applied to BP^20–22^, Schizoaffective Disorder^23^, Schizophrenia^21^, and Major Depression Disorder^24^, among other traits. PRS have also demonstrated a shared genetic risk factors among many of these disorders^25,26^.

The primary aim of the present study is to examine the genetic relationship between genetic risk for anxiety disorders and anxiety comorbidity in bipolar disorder. We aim to answer the question of whether anxiety comorbidity in bipolar disorder reflects distinct genetic risk factors, such that this comorbidity occurs if an individual has high genetic liability for both bipolar disorder and anxiety disorder. Alternatively, this comorbidity may reflect an alternative clinical manifestation (pleiotropy) of the known genetic risk for bipolar disorder. Our secondary research question is whether genetic risk for anxiety contributes to the increase in suicide attempts among people with comorbid bipolar and anxiety disorders. The results shed light on the mechanisms that underlie variable clinical presentations of BP and might help inform clinical management of patients with comorbid BP and anxiety disorders.

## Subjects and methods

### Sample description

The present study used existing data from five independent samples: First, a GWAS of lifetime anxiety disorder undertaken in the UK Biobank^18^, referred to herein as the “discovery sample”. We also include three additional samples; the Genetic Association Information Network (GAIN) bipolar, the Translational Genomics Research Institute (TGEN), and the Swedish Bipolar Disorder Cohort (SWEBIC), which we combined and referred to herein as the “target samples.” Ascertainment, diagnosis, and genotyping of these samples have been previously described^18,27,28^. Further details of the target samples are presented in Table 1. Written informed consent was obtained from all participants. Each study was approved by a local Ethics Committee.

**Table 1:** Sample description.

Sample	Target			
Study name	GAIN	TGEN	SWEBIC	Combined
Sample Size	528	1110	1731	3369
Female (%)	298 (56)	733 (66)	1067 (61)	2098 (62)
Age at BP onset (SD)	18.5 (9)	18.5 (9)	na	18.5 (9)^e^
Comorbidy				
Any anxiety	192	459	518^c^	1169 (51)
Panic disorder (%)	139 (72)	304 (66)	425 (82)	868 (74)
Agoraphobia (%)	69 (36)	169 (37)	na	238 (20)
Specific phobia (%)	55 (29)	167 (36)	na	222 (19)
Social anxiety (%)	56 (29)	169 (37)	na	225 (19)
Other/unspecified anxiety disorder	na	na	93 (18)	105 (8)
Suicide attempt (%)	257^a^ (49)	518^b^ (48)	615^d^ (36)	1390 (42

*SA* suicide attempt, *na* not recorded/available.

^a^Recorded in 527 subjects.

^b^Assessed in 1068 subjects.

^c^Assessed in 636 subjects.

^d^Assessed in 1719 subjects.

^e^Age at BP onset was available only for 1595 subjects (GAIN and TGEN samples).

The discovery sample comprised the largest available GWAS on anxiety disorders, including individuals of western European-ancestry who took part in the UK Biobank online mental health follow-up questionnaire. This sample represented people reporting a lifetime diagnosis by a professional of panic disorder (PD), agoraphobia, social phobia (SP), social anxiety disorder (SAD), or generalized anxiety disorder (GAD)^18^.

### Target sample: GAIN/TGEN/SWEBIC

The GAIN, TGEN, and SWEBIC samples included 3369 individuals of European ancestry assessed by a comprehensive psychiatric interview. All individuals included in the present analysis had received a final DSM-III-R/IV diagnosis of Bipolar I (BPI) or Schizoaffective disorder—bipolar type (SA-BP).

The GAIN and TGEN participants (*N* = 1638) were assessed by a comprehensive psychiatric interview^29^ supplemented by family informant and medical record data. Phenotypic data was harmonized and compiled within the Bipolar Disorder Phenome Database^30^. Four individuals were excluded owing to missing genotype or phenotype data.

The SWEBIC participants (*N* = 1731) were assessed according to the DSM-IV criteria. Genotype data have been previously reported^31^.

### Phenotypes

#### Anxiety comorbidity

Anxiety comorbidity was defined as a lifetime diagnoses of PD, Agoraphobia, SP, SAD, and/or GAD occurring before or after a lifetime diagnosis of BP as assessed during comprehensive psychiatric interview described above.

#### Suicidal attempts

Suicide attempt was defined as a lifetime self-report of self-harm with lethal intent, reported during a structured interview according to previous studies^32^.

#### Polygenic risk score (PRS)

PRS for anxiety were calculated using summary statistics from the UK Biobank discovery sample^18^. We followed the guide for genomic profile risk scoring (Box 1 of reference^25^) to calculate the PRS. In the discovery sample, *p* value informed pruning of correlated SNPs was done from summary statistics by use of LD-based clumping (*r*^2^ threshold < 0.25 across a 500 kilobase window) as implemented in PLINK^33^, using the 1000 Genomes European-ancestry sample (excluding Finns) as a reference panel. The pruned SNPs were used to compute the anxiety PRS in the target samples based on 65,415 shared SNPs. Alleles were weighted by the effect sizes from the discovery sample (log[OR]), including all SNPs that were significant at *p* < 0.5.

We used the same approach to calculate a BP PRS in the target samples, based on summary statistics available from a recently-published BP GWAS^34^. *P* value threshold from the discovery sample was set to 0.2, since this captured the most phenotypic variance.

PRS were standardized using means and SDs from the respective distributions.

BP-PRS showed a small but significant correlation with anxiety-PRS (Pearson *r* = 0.07, *p* < 0.0001).

#### Statistical methods

Association between anxiety PRS and phenotypic information was tested by logistic regression, as implemented in SAS vs. 9.4. Population stratification was corrected with ancestry principal components analysis (PC) based on the variance-standardized relationship matrix in Plink^33^, using the first 5 PCs as covariates. Sex was used as a covariate for anxiety comorbidity, since we observed an association between Anxiety and Sex in the target (*p* < 0.001) sample, consistent with previous findings^8,9^. In addition, the source study (GAIN,TGEN, SWEBIC) was included as a covariate to control for any batch effects.

PRS association *p* values were Bonferroni-corrected for four different tests: Anxiety PRS and BP PRS versus comorbid anxiety or suicide attempts (*p* < 0.0125). Only one *p* value threshold was used for calculating PRS in each of the discovery samples (*p* < 0.5 for anxiety PRS, *p* < 0.2 for bipolar PRS, as noted above).

#### Power calculations

The power of PRS was carried out in AVENGEME^35,36^. The proportion of phenotypic variance explained by common SNPs was estimated from the target sample. The power of anxiety PRS and BP PRS to predict anxiety on BP sample was 90 and 74%, considering the h^2^SNP for anxiety (12%) obtained from the discovery sample^18^. The power of anxiety PRS and BP PRS to detect suicide attempt was 91 and 76%, respectively, given an h^2^SNP of 10% for suicide attempt^32^.

## Results

### Sample characteristics

A total of 3369 patients with a diagnosis of BPI or SA-BP, from 3 different studies, comprised the target sample (Table 1). The studies were similar in terms of European ancestry and sex ratio. A lifetime diagnosis of any anxiety disorder (“Any Anxiety”) was present in 51.6% of the target sample (Table 1). 41.92% of the target sample reported (*N* = 1390) one or more suicide attempts (Table 1).

### Anxiety comorbidity

**Fig. 1:**
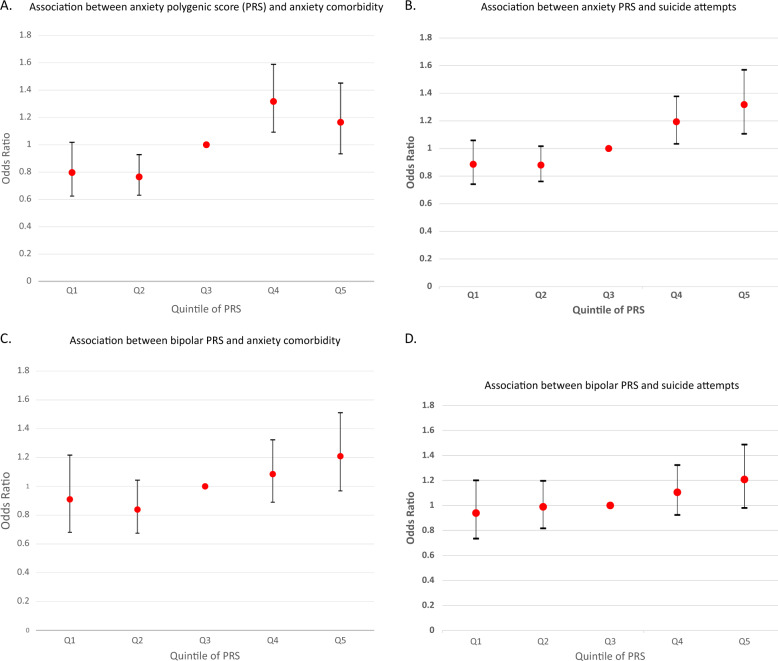
Association of anxiety or bipolar disorder polygenic scores with anxiety comorbidity and suicide attempts. **a** The association between anxiety polygenic score (PRS) and anxiety comorbidity in patients with bipolar disorder. Anxiety comorbidity was evaluated as a categorical phenotype (“Any Anxiety”). **b** The association between anxiety PRS and suicide attempts in patients with bipolar disorder. **c** The association between bipolar PRS and anxiety comorbidity in patients with bipolar disorer. **d** The association between bipolar PRS and suicide attempts in patients with bipolar disorder. Logistic regression results are reported as odds ratios (OR). 95% confidence intervals (CI) are also shown. The PRS data are distributed in quintile.

Anxiety-PRS was significantly and positively associated with “Any Anxiety” in the target sample (Fig. 1a). Each unit increase in anxiety-PRS led to a 15.3% increase in the odds of a comorbid anxiety disorder (OR = 1.153, 95% CI: 1.048–1.269, *p* = 0.0034, Nagelkerke’s *R*^2^ = 0.08).

In contrast, we detected no significant main effect of BP PRS on anxiety comorbidity in the target sample (Fig. 1c) (OR = 1.118, 95% CI: 1.000–1.248, *p* = ns, Nagelkerke’s *R*^2^ = 0.05).

### Suicidal behavior

Anxiety PRS was significantly and positively associated with suicide attempts (SA) in the target sample (Fig. 1b) (OR = 1.106, 95% CI: 1.030–1.189, *p* = 0.0055, Nagelkerke’s *R*^2^ = 0.06). In contrast, we detected no significant association between BP PRS and SA in the target sample (OR = 1.078, 95% CI: 0.998–1.164, *p* = ns, Nagelkerke’s *R*^2^ = 0.02) (Fig. 1d).

## Discussion

To our knowledge, this is the first study to address the impact of genetic risk for anxiety on the clinical course of BP and to provide evidence for a molecular genetic distinction between bipolar disorder with and without comorbid anxiety. The results suggest that bipolar disorder with comorbid anxiety reflects a dual burden of bipolar and anxiety-related genes. Clinical approaches that address this dual genetic burden may help improve outcomes in people living with comorbid bipolar and anxiety disorders.

There were two main findings. First, anxiety disorder comorbidity in bipolar disorder was associated with anxiety PRS in the samples we studied. Second, anxiety PRS was also associated with suicide attempts in BP.

In previous population-based studies, about 60% of bipolar probands met criteria for comorbid anxiety disorders^1,2,37^. In the present study, we found that anxiety risk alleles play a significant role in this comorbidity. Our findings thus contribute to heavily-debated questions concerning the nosologic relationship between mood and anxiety disorders. Our data support the view that anxiety comorbidity in BP is due, at least in part, to the same common genetic risk variants as anxiety in general, while bipolar risk alleles do not. This finding is consistent with the known low genetic correlations between bipolar disorder and anxiety-related traits^38^, which suggests that the disorders share few genetic risk factors.

A second important result arising from the polygenic scoring analyses is the association between anxiety PRS and suicidal behavior. In our target sample we observed a positive association between suicide attempts and genetic risk for anxiety in individuals with a diagnosis of bipolar disorder. These results are consistent with the increased rates of suicidal behavior in people with anxiety disorders reported by population-based studies^11^ and call attention to the importance of monitoring suicide risk in people with BP and comorbid anxiety.

Our results show that anxiety PRS accounts for only a small proportion of overall anxiety comorbidity in BP, so other factors may be involved. One factor could be assortative mating where people with BP are more likely to partner with people with anxiety disorders, leading to increased comorbidity in the offspring. However, Nordsletten et al. have reported a maximal rate of assortative mating between anxiety and Bipolar I of 18%^39^. There is also a small but significant genetic correlation between anxiety and BP, which suggests that BP risk alleles may also contribute to comorbid anxiety^18^. Correlated nongenetic risks may also contribute to comorbidiy. Nongenetic risk factors that might contribute to both BD and anxiety disorders include social isolation, unstable relationships, socioeconomic disadvantage, and traumatic life events^40^. Some anxiety comorbidity may also arise as a complication of BD or its treatment. For example, according to the staging model^41^, anxiety may manifests as a residual symptom following an acute mood episode.

This study should be viewed in the light of several limitations. This is a cross-sectional study, that relied upon retrospective reports. Although the best-estimate diagnosis procedure considers convergent data from family informants and medical records, data on GAD was not available, and the results may underestimate any association between anxiety PGS and the full range of comorbid anxiety disorders. The target sample was underpowered to detect association between BP-PRS with both comorbid anxiety and suicide attempt, so the failure to detect a significant result in this study does not rule out a contribution of BP risk alleles to those trait. Moreover, the overall direction of the relationship between comorbid anxiety or suicide attempt is similar for both ANX-PRS and BP-PRS (Fig. 1c, d), suggesting that a larger target sample may uncover significant associations with BP-PRS. While we used the largest published BP GWAS available, most controls were not formally screened for anxiety. This would not create a false-positive result, but would further reduce the power of a BP-based PRS to detect anxiety in another sample. The results should be considered preliminary until replicated in an independent sample. The lack of a replication sample here reflects the scarcity of available samples worldwide that have been fully characterized for both BP and anxiety. Anxiety-PRS indexes only a small proportion of the variance in anxiety disorder risk. This is an inherent weakness with the PRS method but is expected to improve with increased size of the discovery samples. A further limitation of this study is the reliance of the anxiety sumstats on self-report of a past diagnosis made by an unknown professional or retrospective recall of lifetime symptoms, both of which may contain error. However, we note that Purves et al.^18^ report high genetic correlation between these summary statistics and several other anxiety phenotypes, reassuring us that this case selection approach has utility.

In conclusion, anxiety and suicidal behavior in bipolar disorder are influenced by genetic risk factors involved in anxiety disorders. Patients with comorbid bipolar and anxiety disorders thus carry a dual genetic burden, suggesting the need for clinical approaches that address both disorders. More research is needed to understand the interplay between genetic and non-genetic influences on the clinical presentation and course of BP. Better powered discovery samples for both BP and anxiety will be needed to further elucidate this relationship.
